# A fully-automated statistical method for characterization of flow artifact presence in cardiac MRI

**DOI:** 10.1186/1532-429X-13-S1-P45

**Published:** 2011-02-02

**Authors:** Sotirios A Tsaftaris, Xiangzhi Zhou, Rohan Dharmakumar

**Affiliations:** 1 Northwestern University, Evanston, IL, USA; 2 Northwestern University, Chicago, IL, USA

## Introduction

Flow artifacts in MR images can appear as ghosts within and outside the body cavity. Current approaches for optimizing sequences for suppressing such artifacts rely on expert scoring or on semi-automated methods for evaluation.

## Purpose

To test a fully-automated statistical image-processing method that can quantify the presence of flow artifacts. The method was evaluated against expert scoring in the setting of cine blood-oxygen-level-dependent (BOLD) MRI with different SSFP imaging strategies.

## Methods

### Imaging studies

Six healthy dogs were studied in a Siemens 1.5T scanner using three breath-held 2D SSFP cine sequences (Table 1) in the basal position where flow artifacts are most pronounced. Shared scan parameters: spatial_resolution=1.2x1.2x5mm3, flip_angle=70°, temporal_resolution=10-12ms, no parallel imaging.

**Table 1:** 2D cine SSFP Imaging sequences and parameters used. Shared parameters in text.

Sequence	BOLD sensitivity	Flow artifacts	TR	BW	Flow compensation
A	negligible	minimal	3.5	930	no
B	optimal	significant	6.2	239	no
C	optimal	reduced	6.2	930	yes

### Image processing

Each cine stack I(t) (t denotes trigger time) was loaded in Matlab and the per-pixel mean (I_A_) and variance (I_V_) were found across t. Initialized with a rectangle the size of I_A_, a contour was evolved, using a level-set approach, until it converged to the body-air interface, providing a binary mask *M* (M=1 for air). Image I_R_=I_V_/(I_A_)^2^ (pixel-wise division) was calculated and all values of I_R_ in air (M=1) were collected to estimate the excess kurtosis (γ) of their distribution *H*. The metric Q_K_=γ was used to quantify the presence of flow artifacts.

### Data analysis

Three expert reviewers, blinded to sequence type, scored 16 studies for the presence of ghosts [1(least) to 5(most)]. ANOVA was used to test for differences in scores/metric among sequences. Q_K_ was correlated with the reviewers’ median choice (Q_H_) to assess agreement.

## Results

**Figure 1:**
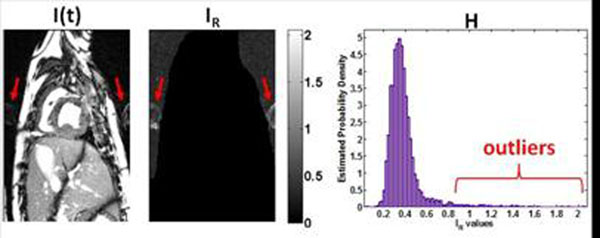
Images from a canine study acquired with sequence B, showing the presence of artifacts and steps of the proposed method. Left: a systolic image (22^nd^ frame in 55 total frames) of the study, showing optimal BOLD sensitivity and significant flow artifacts. Middle: The I_R_ image as defined in the methods indicating that artifact information is retained (only pixels in air are shown as found by the level-set segmentation): the arrows point to artifacts. Right: The distribution (H) of the I_R_ values in air: bracket indicates I_R_ values from ghost artifact regions (see arrows in middle image) that act as outliers. Due to the presence of these outliers the excess kurtosis for this stack was y=Q_K_=21.

**Figure 2:**
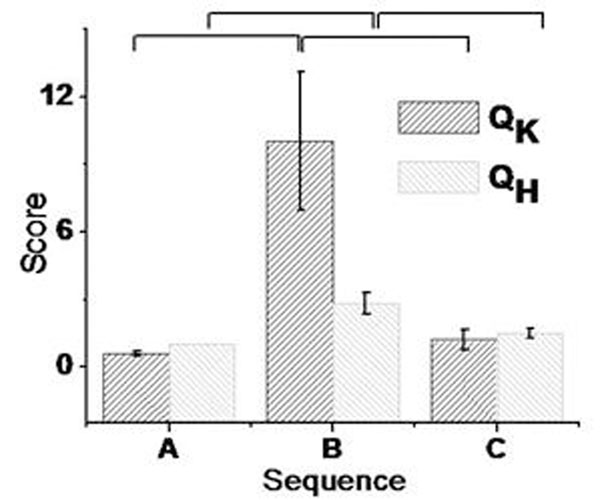
Bar plots (mean ± standard error) for Q_K_ (derived using the kurtosis-based method) and Q_H_ (the experts median choice per study) grouped by sequence type. Intervals on top indicate statistical significance of individual comparisons (P<0.05). Kruskal-Wallis ANOVA with Tukey-Post-Hoc analysis of Q_K_ and expert scores identified a difference in the presence of ghost artifacts in images acquired with sequences A and B or B and C.

Fig. 1 shows a representative case from a study acquired with sequence B. Fig. 2 shows bar plots of Q_K_ values and scores (Q_H_) for each imaging sequence. Correlation coefficient between Q_K_ and Q_H_ was 0.7 (R^2^=0.49;P<0.01).

## Conclusions

Statistical comparisons of Q_K_ scores identified a difference in the presence of ghost artifacts among the three sequences in full agreement with expert findings. This indicates that this kurtosis-based method can assess the variability of artifact presence in a stack without the need to process each image separately. In contrast to other methods, the proposed approach uses high order statistics (kurtosis) of background pixels to estimate ghost presence and is robust against (coil) bias due to the division with per-pixel mean image I_A_. Although further studies are needed, the proposed approach may be useful in readily assessing image quality in a clinical/research setting in an unbiased and fully-automated manner.

